# Isolation, biochemical and molecular identification of *Nocardia* species among TB suspects in northeastern, Tanzania; a forgotten or neglected threat?

**DOI:** 10.1186/s12879-017-2520-8

**Published:** 2017-06-08

**Authors:** Abubakar S. Hoza, Sayoki G.S. Mfinanga, Irmgard Moser, Brigitte König

**Affiliations:** 1 Department of Medical Microbiology and Epidemiology of Infectious Diseases, Faculty of Medicine, University of Leipzig, Liebig Str. 21, 04103 Leipzig, Germany; 2 Department of Microbiology, Parasitology and Immunology, College of Veterinary and Medical Sciences, Sokoine University of Agriculture, P. O. Box, 3019 Morogoro, Tanzania; 3 National Institute for Medical Research, Muhimbili Medical Research Centre, P. O. Box, 3436 Dar es Salaam, Tanzania; 4 Friedrich Loeffler Institut, Institute of Molecular Pathogenesis, Naumburger Str. 96a, 07743 Jena, Germany

**Keywords:** *Nocardia*, Tuberculosis, Biochemical, Molecular, Northeastern, Tanzania

## Abstract

**Background:**

Pulmonary nocardiosis mimic pulmonary tuberculosis in most clinical and radiological manifestations. In Tanzania, where tuberculosis is one of the major public health threat clinical impact of nocardiosis as the cause of the human disease remains unknown. The objective of the present study was to isolate and identify *Nocardia* isolates recovered from TB suspects in Northeastern, Tanzania by using biochemical and molecular methods.

**Methods:**

The study involved 744 sputum samples collected from 372 TB suspects from four periphery diagnostic centers ﻿in﻿ Northeastern, Tanzania. Twenty patients were diagnosed as having presumptively *Nocardia* infections based on microscopic, cultural characteristics and biomèrieux ID 32C Yeast Identification system and confirmed using 16S rRNA and *hsp*65 gene specific primers for *Nocardia* species and sequencing.

**Results:**

Biochemically, the majority of the isolates were *N. asteroides* (*n* = 8/20, 40%), *N. brasiliensis* (*n* = 4/20, 20%), *N. farcinica* (*n* = 3/20, 15%), *N. nova* (*n* = 1/20, 5%). Other aerobic actinomycetales included *Streptomyces cyanescens* (*n* = 2/20, 10%), *Streptomyces griseus*, *Actinomadura madurae* each (*n* = 1/20, 5%). Results of 16S rRNA and *hsp*65 sequencing were concordant in 15/17 (88. 2%) isolates and discordant in 2/17 (11.8%) isolates. Majority of the isolates belonged to *N. cyriacigeorgica* and *N. farcinica,* four (23.5%) each.

**Conclusions:**

Our findings suggest that *Nocardia* species may be an important cause of pulmonary nocardiosis that is underdiagnosed or ignored. This underscores needs to consider pulmonary nocardiosis as a differential diagnosis when there is a failure of anti-TB therapy and as a possible cause of human infections.

**Electronic supplementary material:**

The online version of this article (doi:10.1186/s12879-017-2520-8) contains supplementary material, which is available to authorized users.

## Background

Members of genus *Nocardia* are characteristically gram-positive, weakly acid-fast, strictly aerobic, filamentous branching bacilli that fragment into rod or coccoid shaped forms. *Nocardia* species are ubiquitous environmental bacteria capable of causing opportunistic infections in both human and animals [1, 2]. *Nocardia* species are increasingly isolated as infectious agents in immunocompromised patients, and at times, even among healthy individuals [3], causing infections ranging from pulmonary, cutaneous and subcutaneous human diseases [4].

The most commonly isolated species in human include *N*. *asteroides, N*. *farcinica*, *N. cyiriacigeorgica*, *N.nova*, *N. brasiliensis* [5–7]. Pulmonary nocardiosis has been reported in patients with debilitating conditions, such as those with organ transplants, diabetes mellitus, leukemia, and other malignancies [8].

The incidence of nocardiosis varies geographically according to a number of factors, like the prevalence of HIV infections, transplants, cancer, climate as well as socio-economic status, and laboratory capacity for *Nocardia* species detection and identification.

Increased incidence of human nocardiosis may be attributed to the wide use of immunosuppressive drugs, improved diagnostic tests, and increased awareness among microbiologists and health professionals. Nonetheless, in many developing countries where other chronic lung diseases, particularly TB, are prevalent, *Nocardia* species are either missed or misidentified during diagnosis [9, 10].

Accurate detection and identification of *Nocardia* species have become increasingly important for prediction of antimicrobial susceptibility since different species have emerged in terms of their virulence and epidemiology. Furthermore, prompt and timely species identification can significantly influence the choice of therapy.

Apart from Gram and modified acid-fast staining, identification of *Nocardia* species depends largely on biochemical tests and cellular fatty acid analysis, which have proved to be laborious with long turnaround time, and less definitive.

Several molecular identification methods have been successfully employed to identify and characterize *Nocardia* species. Multilocus sequence analysis (MLSA) of 16S rRNA, the 65-kDa heat shock protein (*hsp*65), gyrase B of the ß subunit of DNA topoisomerase (*gyrB*), subunit A of Sec A preprotein translocase (*secA1*) and RNA polymerase (*rpoB*) have previously been used to identify *Nocardia* species [11–13]. However, 16S rRNA gene sequencing, which is the most often used system of bacterial identification, cannot discriminate many species [14], therefore identification based on the basis of the DNA sequence of a single housekeeping gene is hindered by stochastic genetic variation as well as horizontal gene transfer and recombination [15].

Pulmonary nocardiosis mimics pulmonary tuberculosis in both clinical manifestations, being chronic in nature and radiological characteristics makes it difficult to differentiate from *M. tuberculosis* and may as well be often wrongly treated with anti-TB drugs [16, 17]. Patients might also be confused with other chronic lung infections such as invasive fungal infection [18, 19].

In Tanzania, where tuberculosis is one of the major public health threats and a third leading cause of adult morbidity and mortality after Malaria and HIV/AIDS; clinical reports of *Nocardia* are rare if found at all. Moreover, the knowledge, as well as the clinical impact of nocardiosis, is unknown, suggesting that infections due to this genus may be underdiagnosed and/or neglected as a cause of human diseases.

Due to the lack of information on pulmonary nocardiosis in Tanzania, the objective of the present study was to isolate and identify *Nocardia* isolates recovered from TB suspects in Northeastern, Tanzania by using biochemical and molecular methods.

## Methods

### Collection and decontamination of clinical specimens

Three hundred and seventy-two (*n* = 372) self-presented TB suspects were included in this study after presumptively diagnosed as pulmonary tuberculosis cases by clinical symptoms and microscopically by acid-fast Ziehl-Neelsen (ZN) and or fluorescence staining method from four peripheral diagnostic centers (PDCs) namely Makorora health centre, Ngamiani health centre, Bombo regional hospital and Muheza designated district hospital in Northeastern, Tanzania. From each patient, two sputum specimens were collected one spot during the first visit at the respective TB facilities and one early morning specimen. The specimens were decontaminated by N-acely L-cystiene (NALC) sodium hydroxide (NaOH) method as described previously [20]. Decontaminated specimens were concentrated by centrifugation at 3000×g for 20 min after discarding the supernatant the sediments were resuspended by adding 1.5 ml of phosphate buffered saline (PBS). All procedures were carried out in a certified level II biosafety cabinet.

### Culture and identification of the isolates

Two drops of the centrifuged sediments were inoculated on Löwestein-Jensen (L-J) and incubated at 37 °C for 8 weeks. Subsequently, 500 μl of each specimen was inoculated in a BacT/Alert bottle, incubated in the BacT/Alert® 3D system for 8 weeks. The sample was considered negative when no bacterial growth or positive signal from the BacT/Alert instrument was observed after 8 weeks incubation. Positive cultures were stained by ZN for the confirmation of the acid-fast bacilli (AFB). Confirmation of *M. tuberculosis* was performed by using GenoType® MTBC (Hain Life science GmbH, Nehren, Germany) and GenoType® Mycobacterium CM/AS assay (Hain Life science GmbH, Nehren, Germany) for the detection of common and accessory nontuberculous mycobacteria (NTM).

Negative cultures for AFB were cultured on blood agar (BA) and chocolate agar plates and incubated at 37 °C for 2 to 4 days. Isolates were diagnosed as *Nocardia* based on the presence of non-acid-fast branching filamentous bacilli using Gram and modified Kinyoun staining, as well as their colony morphology.

### Biochemical identification

The BioMèrieux ID 32C yeast identification system was used to identify the presumptively 20 *Nocardia* isolates diagnosed in this study. All procedures followed manufacturer’s recommendations. Briefly, test isolates were grown on brain heart infusion agar or chocolate agar plates at 35 °C. A Mc Farland no.4 was prepared in a standard physiological saline. One ml of the suspension was inoculated into ID 32C medium, and 3 drops were dispensed into each well of the strip using an automated dispenser ATB Vitek® 1574 (biomèrieux). The strips were incubated at 35 °C for 7 days in a sealed container to avoid evaporation. The strips were read to give an eight-digit profile, as per manufacturer’s instruction.

### DNA extraction

DNA extraction was performed by using Ultraclean® Microbial DNA isolation kit (MOBIO Laboratories, Inc.) following manufacturer’s instructions, after an initial heat inactivation of a loopful colony suspended in 500 μl-distilled water in a 2 ml Eppendorf tube then incubation at 95 °C for 20 min followed by centrifugation at 15,000 x g for 5 min at room temperature (r.m.t). 50 μl of collected DNA was then stored at -20 °C until use. *M. tuberculosis* reference strain H37Rv genomic DNA and distilled water were used as positive and negative controls respectively.

### PCR for 16S rRNA

A 606-bp fragment of the 16S rRNA gene specific for *Nocardia* species was amplified with primers Noc1 (5′-GCTTAACACATGCAAGTCG-3′) (positions 46 to 64, *Escherichia coli* numbering system) and Noc2 (5′-GAATTCCAGTCTCCCCTG-3′) (positions 663 to 680, *E. coli* numbering system) [1]. PCR reaction was performed in a 25 μl volume with master mix contained a final concentration of 5 U *Taq* DNA polymerase (Applied Biosystems), 10× buffer, 10 mM dNTPs mix, 100 pmol/μl of each primer, 25 mM MgCl_2,_ and 100 ng template DNA. PCR amplification was carried out with an initial denaturation of 94 °C for 5 min, followed by 40 cycles of denaturation at 94 °C for 1 min, annealing at 58 °C for 1 min, extension at 72 °C. Then followed by a final extension at 72 °C for 5 min. PCR products were purified with Qiagen PCR purification kit (Qiagen, CA, USA) following manufacturer’s recommendations and were submitted for sequencing at the Department of Biochemistry, University of Leipzig, Germany.

### PCR specific for *hsp*65 gene

A 440-bp fragment of the *hsp*65 gene encoding the 65 kDa heat shock protein was amplified using (TB11, 5′-ACCAACGATGGTGTGTCCAT-3′) and (TB12, 5′-CTTGTCGAACCGCATACCCT-3′) oligonucleotide primers [21]. PCR was performed in a final volume of 25 μl (5 U of *Taq* DNA polymerase (Applied Biosystems); 10× buffer, 25 mM MgCl2, 10 mM dNTPs mix, 100 pmol of each primer) with 10 μl of DNA template. Amplification was carried out in a thermal cycler (Applied Biosystems). Amplification cycles included an initial denaturation at 94 °C for 5 min, followed by 35 cycles (denaturation 94 °C for 1 min, annealing at 55 °C for 1 min, extension at 72 °C for 1 min) and a final extension at 72 °C for 10 min. For sequencing the PCR product of the *hsp*65 gene were purified by Qiagen PCR purification kit as recommended by the manufacturer (Qiagen, CA, USA) and submitted for sequencing at the Department of Biochemistry, University of Leipzig, Germany). Same primers used for PCR served for sequencing of the forward and reverse fragments.

### Sequencing of 16S rRNA and *hsp*65 PCR product fragments

PCR products were purified and sequenced on both strands. The resulting 16S rRNA and *hsp*65 sequences of the 20 isolates were analyzed using Codon Code Aligner software Version 5.1.5 (http://www.codoncode.com/aligner/). 16S rRNA gene sequences were compared with the corresponding sequences of the *Nocardia* species in the GenBank database (www.ncbi.nlm.nih.gov) and LeBIBI^QBPP^ database using the SSU-rDNA-16S_TS-stringent [22] where there were disparities the LeBIBI^QBPP^ assigned species name was adopted; for the *hsp*65 gene only the GenBank database was used. For the comparison with GenBank database the Basic Local Alignment Sequencing Tool for nucleotide sequence queries (BLASTN) was used. The GenBank query type strain or culture collection strain with the highest score was downloaded and compared to the subject sequences and the percentage similarity was then determined for each strain. Strains with greater than 99% homology to a single type strain were considered definitive for identification to species level.

### Ethical clearance

The protocol for this study was reviewed and approved by the Ethical Committee of the National Institute for Medical Research (NIMR) (Ref. No. NIMR/HQ/R.8a/Vol.IX/1401), Dar es Salaam, Tanzania. Written informed consent was obtained from the patients or relative of the patients, where the patients could not read and write. Ethical approval was obtained for the secondary use of the study data from the same ethical committee.

## Results

### Detection of *Mycobacteria* and *Nocardia* species

Of 372 decontaminated samples sixty-five (*n* = 65) were AFB positive by ZN staining, whereas one-hundred thirty-seven (*n* = 137) including the AFB positive were culture positive by either L-J or BacT/Alert 3D system. Two hundred and two (*n* = 202) samples were both AFB and culture negative and 33 samples were contaminated. A specimen was considered as contaminated when there was a growth of non-mycobacterial organisms in either L-J or BacT/Alert bottle. The purity of all fluid cultures by plating them on blood agar and chocolate agar plates and re-examining individual specimen by ZN-stain.

**Fig. 1:**
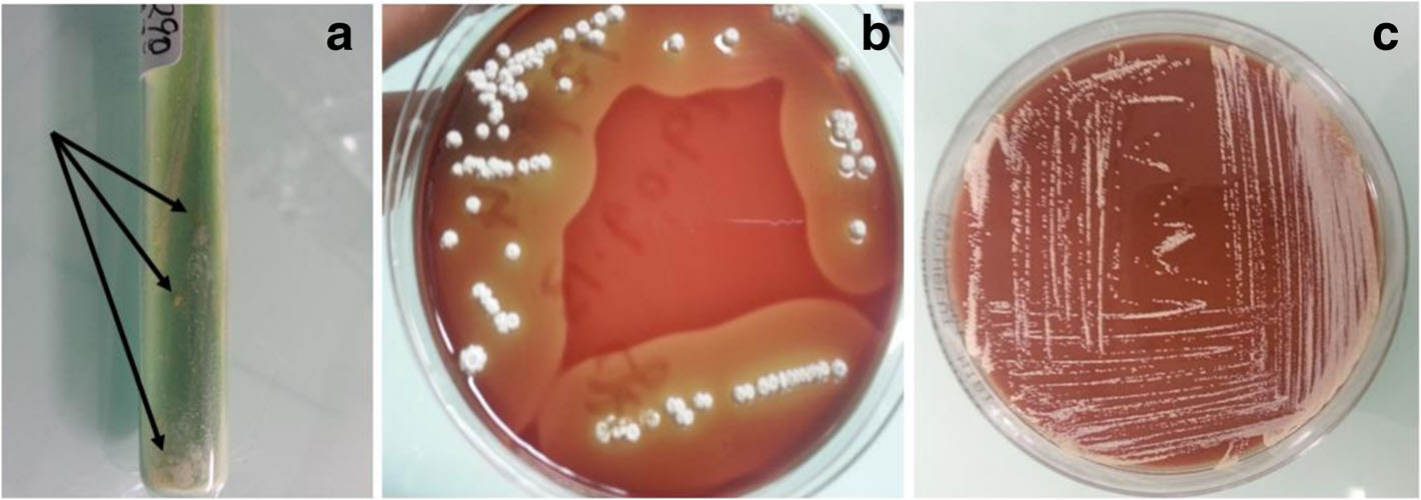
Growth of *Nocardia* spp. isolated from TB suspects on three different media used in this study (**a**) LJ medium (see arrows), (**b**) Blood agar plate and (**c**) Chocolate agar plate

Eighty-one of the AFB isolates were confirmed to be *M. tuberculosis* using GenoType® MTBC (Hain Life science GmbH, Nehren, Germany) and 36 isolates were confirmed as NTM by GenoType® Mycobacterium CM/AS (Hain Life science GmbH, Nehren, Germany). Twenty (5.4%) out of 372 TB suspects which are subject of this manuscript were presumptively diagnosed as having *Nocardia* infection. Of these nine (9/20) isolates were among the 137 isolates mistakenly recorded as AFB positive at the PDCs (see Additional file 1). Diagnosis of *Nocardia* species based on the presence of non-acid-fast branching filamentous bacilli using Gram and modified ZN staining, as well as their colony morphology on LJ, BA and Chocolate agar (Fig. 1). Eleven isolates grew well on LJ media, and all isolates grew on both BA and Chocolate agar.

### Profiles of isolates obtained by using the biomèrieux ID 32C yeast identification system

Twenty isolates presumptively diagnosed as *Nocardia* species in this study were tested by the Biomèrieux ID 32C Yeast Identification system and results obtained showed that majority of the isolates were *N.asteroides* (*n* = 8/20, 40%), *N. brasiliensis* (*n* = 4/20, 20%), *N. farcinica* (*n* = 3/20, 15%), *N. nova* (*n* = 1/20, 5%). Other aerobic actinomycetes identified included *Streptomyces cyanescens* (*n* = 2/20, 10%), *Streptomyces griseus*, and *Actinomadura madurae* each (*n* = 1/20, 5%).The profiles of the twenty isolates obtained using the biomèrieux ID 32C yeast identification system in this study are shown in Table 1.

**Table 1:** Profiles of different Nocardia species and other aerobic Actinomycetes identified using the biomèrieux ID 32 C Yeast Identification system in this study

Isolate	Profile	Organism
1	37,735,522	*Streptomyces cyanescens*
2	37,735,522	*Streptomyces cyanescens*
3	37,516,506	*S. griseus*
4	75,112,246	*A. madurae*
5	71,414,140	*N. brasiliensis*
6	20,004,004	*N. nova*
7	26,420,142	*N. asteroides*
8	10,000,304	*N. farcinica*
9	61,400,304	*N. farcinica*
10	00000106	*N. asteroides*
11	00040100	*N. asteroides*
12	20,000,100	*N. asteroides*
13	00000104	*N. asteroides*
14	31,014,100	*N. brasiliensis*
15	31,414,100	*N. brasiliensis*
16	20,000,401	*N. asteroides*
17	20,000,100	*N. asteroides*
18	10,000,204	*N. farcinica*
19	71,414,140	*N. brasiliensis*
20	00040100	*N. asteroides*

### Identification of *Nocardia* species by 16S rRNA and *hsp*65 gene sequencing

Since *Streptomyces* species are difficult to identify by the biomèrieux ID 32C Yeast Identification system because they give a less distinct profile pattern than *Nocardia* isolates [23], all 20 isolates tested by this method for the 16S rRNA were analysed using the LeBIBI^QBPP^ a web tool [22] and the GenBank database, where there was disparities between the two databases the species name assigned by the former was adopted, and for sequences without the closest proximal cluster in LeBIBI^QBPP^, the closest sequence was identified based on the tree determination. Sequencing of *hsp*65 gene revealed that all 17 confirmed *Nocardia* isolates had closest species similarity identity score of >99% with type or culture collection strain in the database on one hand, and on the other the 16S rRNA sequencing, thirteen (*n* = 13) isolates had closest species belonging to the proximal clusters in the LeBIBI^QBPP^ database with four (*n* = 4) isolates not belonging to the closest proximal cluster, hence their identification was based on the tree determination. Generally, *hsp*65 and 16S rRNA gene sequencing were concordant in 15 out of 17 (88.2%) isolates and discordant in two out of 17 (11.8%) isolates. Of the discordant results 16S rRNA identified one isolate as *N. jinanensis* TDQ462650 proximal cluster and another as *N. thailandica* TGQ376186 proximal cluster*,* whereas *hsp*65 identified these isolates as *N. testacea* and *N. cyriacigeorgica* respectively (Table 2). Details of the isolates and their accession numbers are provided in Additional file 1.

**Table 2:** Direct smear microscopy results and identification of different *Nocardia* isolates (*N* = 17) from TB suspects in Northern, Tanzania using Biomèrieux ID 32C Yeast identification System, 16S rRNA and *hsp*65 gene sequencing

Smear microscopy	API profile	Biomèrieux ID 32C yeast identification system	16S rRNA gene	Proximal cluster	*hsp*65 gene	Identity (%)
Sm + ve	37,735,522	*Streptomyces cyanescens*	*N. farcinica*	NA*	*N.farcinica*	99
Sm-ve	71,414,140	*N. brasiliensis*	*N. cyriacigeorgica*	TDQ659904	*N. cyriacigeorgica*	99
Sm-ve	20,004,004	*N. nova*	*N. jinanensis*	TDQ462650	*N. testacea*	100
Sm-ve	26,420,142	*N. asteroides*	*N. farcinica*	NA*	*N.farcinica*	99
Sm-ve	10,000,304	*N. farcinica*	*N. flavorosea*	TAY756552	*N. flavorosea*	99
Sm-ve	61,400,304	*N. farcinica*	*N. farcinica*	TAY756551	*N. farcinica*	99
Sm-ve	00000106	*N. asteroides*	*N. cyriacigeorgica*	TDQ659904	*N. cyriacigeorgica*	99
Sm-ve	00040100	*N. asteroides*	*N. nova*	TGQ376190	*N. nova*	100
Sm + ve	20,000,100	*N. asteroides*	*N. carnea*	TZ36929^b^	*N. sp./flavorosea*	99
Sm + ve	00000104	*N. asteroides*	*N. farcinica*	NA*	*N. farcinica*	99
Sm-ve	31,014,100	*N. brasiliensis*	*N. thailandica*	TGQ376186	*N. cyriacigeorgica*	99
Sm-ve	31,414,100	*N. brasiliensis*	*N. cyriacigeorgica*	TDQ659904	*N. cyriacigeorgica*	99
Sm + ve	20,000,401	*N. asteroides*	*N. testacea*	TAY903612	*N. testacea*	99
Sm-ve	20,000,100	*N. asteroides*	*N. testacea*	TAY903612	*N. testacea*	100
Sm-ve	10,000,204	*N. farcinica*	*N. brevicatena*	TAY756545	*N. brevicatena*	99
Sm-ve	71,414,140	*N. brasiliensis*	*N. flavorosea*	TAY756552	*N.flavorosea*	99
Sm-ve	00040100	*N. asteroides*	*N. cyriacigeorgica*	TDQ659904	*N. cyriacigeorgica*	99

Sm + ve = smear positive; Sm-ve = smear negative, API profile = for the identification of different *Nocardia* species as per ID 32C Yeast identification system, NA* = No acceptable cluster, identification based on tree determination, TZ36929^b^ based on patristic distance

**Table 3:** Clinical data of the 17 patients with Nocardia infections as confirmed by the 16S rRNA gene sequencing

	*N. cyriacigeorgica* (*n* = 4)	*N. farcinica* (*n* = 4)	*N. testacea* (*n* = 2)	*N. flavorosea* (*n* = 2)	*N. nova* (*n* = 1)	*N. jinanensis* (*n* = 1)	*N. thailandica* (*n* = 1)	*N. carnea* (*n* = 1)	*N. brevicatena* (*n* = 1)	Total N (%)
Age (yrs):										
**•** ≤ 35	1	1	2	1	0	0	1	0	1	7 (41.2)
**•** ≥ 35	3	3	0	1	1	1	0	1	0	10 (58.8)
Sex										
**•** Male	2	2	0	2	1	1		0	0	8 (47.1)
**•** Female	2	2	2	0	0	0	1	1	1	9 (52.9)
Clinical symptoms										
**•** Chronic cough ≥2 weeks	4	4	2	2	1	1	1	1	1	17 (100)
**•** Haemoptysis	1	1	0	1	1	0	1	0	0	5 (29.4)
**•** Dyspnoea	3	3	0	1	0	1	0	1	1	10 (58.8)
**•** Night sweat	1	3	1	1	1	1	1	1	1	11 (64.7)
**•** Fever	4	3	2	2	0	1	0	1	1	14 (82.4)
**•** Fatigue	2	2	1	2	0	1	1	1	0	10 (58.8)
**•** Weight loss	1	2	0	1	0	1	0	1	1	7 (41.2)
Concurrent conditions										
**•** Smoking	2	2	0	0	1	0	1	0	0	6 (35.3)
**•** TB**-** Coinfection	0	1	0	0	0	0	0	1	0	2 (11.8)
**•** HIV**-** Coinfection	1	1	0	1	0	0	0	0	0	3 (17.7)
**•** *Nocardia* alone	4	2	2	1	1	1	1	0	1	13 (76.5)
Smear Microscopy										
**•** AFB positive	0	1	0	0	0	0	0	1	0	2 (11.8)
**•** AFB negative	4	3	2	2	1	1	1	0	1	15 (88.2)
Treatment initiated										
**•** Anti-TB drugs	0	1	0	0	0	0	0	1	0	2 (11.8)
**•** Broad-spectrum antibiotics	4	2	1	1	0	1	0	0	1	10 (58.8)
**•** Not treated	1	1	1	1	1	0	0	0	0	5 (29.4)

### Demographic and clinical data of patients diagnosed with *Nocardia* infection

Based on the 16S rRNA and *hsp*65 sequencing results the confirmed 17 cases of *Nocardia* isolates from this study, we revised the demographic and clinical data of all the patients obtained during the study period as shown in Table 3. The clinical symptoms and concurrent conditions show that the proportion of *Nocardia* infection was higher (58.8%) in individuals with ≥35 years of age than among those ≤35 years of age (41.2%), and both sexes are equally affected. The data further showed that nearly all patients presented with clinical syndromes mimicking those of pulmonary tuberculosis. The proportion of individuals with *Nocardia* infection alone was 76.5% (*n* = 13), three (*n* = 3, 17.7%) cases had co-infection with HIV and two (*n* = 2, 11.8%) cases were co-infected with TB and they had commenced anti-TB treatment. Ten (*n* = 10, 58.8%) other cases were initiated with broad-spectrum antibiotic treatment whereas, for five (*n* = 5, 29.4%) cases records showed that they had no treatment whatsoever.

## Discussion

*Nocardia* infections causing both human and animal diseases are increasingly reported owing to improved diagnostics especially in developed settings which have emphasized the need for rapid characterization of clinically isolated *Nocardia* [4, 8, 10, 24, 25]. However, data regarding nocardiosis from resource-poor settings like Tanzania heavily stricken by HIV and TB pandemic are rare if available at all. Such diseases are either underdiagnosed or neglected due to the similarity of clinical and radiological features between pulmonary nocardiosis and pulmonary tuberculosis on one hand and on the other due to poor diagnostic capabilities. In Tanzania where TB is still a major public health threat, anti-TB treatment is initiated based on clinical symptoms, direct smear microscopy and radiological diagnosis where available.

In this study, all 372 TB suspects self-presented at four health facilities were diagnosed based on clinical symptoms and direct smear microscopy by acid-fast staining method at the respective clinics. Eighty-one (*n* = 81, 21.8%) patients were positive for *M. tuberculosis* by culture and confirmed by GenoType® MTBC and 36 (9.7%) patients had NTM infections confirmed by GenoType® Mycobacterium CM/AS. 202/372 patients sputum samples were negative by culture and 33 other samples were contaminated.

Twenty (5.4%) isolates out of 372 TB suspects were presumptively diagnosed as *Nocardia* species based on their colony morphology on different culture media and by microscopic appearance using modified ZN for weak acid-fast bacilli.

Isolates of *Nocardia* species in this study showed good growth on LJ, BA and chocolate agar (Fig. 1). Decontamination of sputum samples with NALC-NaOH in this study did not seem to affect the growth of *Nocardia* species as observed in a previous study [26].

Samples from 17 suspects were presumptively diagnosed as having *Nocardia* infection and three patients had infection with other Actinomycetales. Findings that majority of *Nocardia* species grow well on LJ in this study correspond with findings of other authors [25, 27]. In this scenario, such growth may be confused with that of mycobacteria species, making the diagnosis more complicated. Furthermore, careful microscopic analysis should be thoroughly performed, since these two genera may present difficulties for an inexperienced microscopist [24]. As observed in the previous study [25], where *Nocardia* species were detected by the modified ZN-method for weak acid-fast bacteria, we also detected *Nocardia* species by the ZN-method employed for mycobacteria in this study.

All the *Nocardia* species identified in this study have been reported to be associated with pulmonary nocardiosis in many parts of the world [24, 26–29].

The finding that the biomèrieux ID 32C yeast identification system identified correctly five *Nocardia* isolates to species level and two isolates to least genus level as *Streptomyces* (Table 1) when compared to 16S rRNA and *hsp*65 sequencing results is in agreement with those in a study conducted in Brazil [13]. In the Brazilian study, seven isolates were identified by 31 different phenotypic tests using six identification systems with only two isolates correctly identified by the phenotypic method, compared to multilocus sequence analysis (MLSA) results. However, the biomèrieux ID 32C yeast identification system used in this study appears to be useful in identifying *Nocardia* species and other aerobic actinomycetes as previously determined [23].

The discrepancies between the ID 32C yeast identification system and sequencing results suggest that a careful interpretation of results indicating different *Nocardia* species and other Actinomycetales is needed since such methods are not accurate as shown in Table 2. Moreover, although the ID32C system has been used for identification of many aerobic actinomycetes, it does not, however, give consistent results within a species. There is a greater potential for errors than with 16S rRNA or with *hsp*65 gene sequencing. For example ID32C system present a lot of difficulties in identification of *Nocardia asteroides* complex, this method uses eight-digit profile as those used for identification of yeast, whereas the last four-digits show a consistent correlation between isolates within species, the first four-digits show poor correlation within a species and between species. There is no single profile for each particular isolate and species with this method, hence this variation. Therefore, results of 16S rRNA and *hsp*65 gene sequencing are more reliable than those of the ID32C system. Therefore, molecular identification is necessary for definitive identification of *Nocardia* species [10, 30–33].

Important to note is that the following species: *N. cyriacigeorgica*, *N. farcinica*, *N. brevicatena* and *N. nova* identified by both 16S rRNA and *hsp*65 sequencing in this study were separated from the originally referred *Nocardia asteroides* and later found to be a group of bacteria with a heterozygous pattern of antimicrobial drug susceptibilities [34]. *N asteroides* complex was further separated and reorganized into different species on the basis of drug susceptibility patterns: *Nocardia abscessus*, *Nocardia brevicatena-paucivorans* complex, *Nocardia nova* complex (includes *N nova*, *Nocardia veterana*, *Nocardia africana*, *Nocardia kruczakiae*), *Nocardia transvalensis* complex, *Nocardia farcinica*, and *N asteroides* [5]. Moreover, *Nocardia cyriacigeorgica*, which constitute the majority of *Nocardia* species in this study (*n* = 4, 23.5%) was also differentiated from *N asteroides* and is becoming a more frequently identified clinically significant pathogen [35].

This separation may explain the observed discrepancy between the two methods. While phenotypic identification leads to misidentification, molecular identification can improve the diagnostic accuracy since some molecular targets can present high sequence similarity [2].

Looking into the demographic data of all 17 *Nocardia* cases in this study, it was observed that nine (52.9%) were males and eight (47.1%) were females. Seven (41.1%) were ≤35 years and ten (58.8%) were ≥35 years. This incidence suggests that nocardiosis can occur in both sexes and in different age groups with more or less the same frequency. However, of importance is that nearly all cases of nocardiosis identified in this study reported similar clinical symptoms as those of TB suspects with chronic coughing featuring in all cases (Table 3). This is not surprising since pulmonary nocardiosis is the most common clinical presentation acquired primarily by inhalation. The onset of symptoms can be subacute to more chronic and can include productive or a non-productive cough, shortness of breath, chest pain, hemoptysis, fever, night sweats, weight loss, and progressive fatigue [4].

In this study, 12 patients had *Nocardia* alone; two had co-infection with TB and were AFB positive, whereas three had co-infection with HIV. Similar findings have been reported in other studies [8, 36]. It is important that irrespective of a patient’s immunologic status, the isolation of *Nocardia* from the respiratory tract or another body source should not be regarded as a contaminant or commensal organism [4].

## Conclusion

In conclusion, our study reveals that *Nocardia* species are important causes of pulmonary disease and that may have been underdiagnosed and/or ignored altogether. It is also clear that mycobacterial infections and nocardiosis treatment differ, correct identification of the causative agent is, therefore, critical to avoid treatment failure. *Nocardia* species identified in this study, underscore the need to consider pulmonary nocardiosis as differential diagnosis especially when there is treatment failure with the standard anti-TB therapy, and as a possible cause of human infections.

## Additional file

Additional file 1: Detailed description of the clinical symptoms, concurrent conditions associated with Nocardia infection in Northeastern, Tanzania and the results of Biochemical and Moelcular identification. (XLSX 19 kb)

## Abbreviations

AFB: acid-fast bacilli
BA: blood agar
GenoType® Mycobacterium CM: (for common mycobacteria) and AS (for addition species of mycobacteria)
*gyrB*: gyrase B of the ß subunit of DNA topoisomerase
*hsp*65: 65-kDa heat shock protein
L-J: Löwestein-Jensen
MLSA: Multilocus sequence analysis
MTBC: *Mycobacterium tuberculosis* complex
NALC: N-acely L-cystiene
NaOH: sodium hydroxide
NTM: nontuberculous mycobacteria
PBS: phosphate buffered saline
PDCs: peripheral diagnostic centres
*rpoB*: RNA polymerase
r.m.t: room temperature
*secA1*: subunit A of Sec A preprotein translocase
Z-N: Ziehl-Neelsen
